# Sex-specific associations between the developmental alterations in the pituitary-thyroid hormone axis and thyroid nodules in Chinese euthyroid adults: a community-based cross−sectional study

**DOI:** 10.3389/fendo.2024.1379103

**Published:** 2024-05-10

**Authors:** Ying Li, Genfeng Yu, Nanfang Yao, Siyang Liu, Dongmei Wang, Qintao Ma, Lan Liu, Heng Wan, Jie Shen

**Affiliations:** ^1^ Department of Endocrinology and Metabolism, Shunde Hospital, Southern Medical University (The First People’s Hospital of Shunde, Foshan), Foshan, Guangdong, China; ^2^ School of Nursing, Southern Medical University, Guangzhou, Guangdong, China

**Keywords:** pituitary-thyroid hormone axis, sensitivity to thyroid hormones, thyroid nodules, TFQI, sex-specific

## Abstract

**Background:**

Previous studies have revealed the sex-specific features of pituitary–thyroid hormone (TH) actions and the prevalence of thyroid nodules (TNs) in children and adolescents. However, it was unclear in adults. We aimed to investigate the features of pituitary–TH actions in women and men at different ages, and the associations of thyrotropin (TSH), THs, and central sensitivity to THs indices including the thyroid feedback quantile-based index by FT4 (TFQI_FT4_) and the thyroid feedback quantile-based index by FT3(TFQI_FT3_) with of TNs in Chinese euthyroid adults.

**Methods:**

8771 euthyroid adults from the communities in China were involved. Demographic, behavioral, and anthropometric data were gathered through the questionnaires. Ultrasound was performed to evaluate the TNs. TSH and THs levels were measured. The multivariable logistic regression and multivariable ordinal logistic regression were conducted.

**Results:**

TFQI_FT3_ among both genders, except women aged 43 to 59 years, where it increased slightly. Additionally, there was an age-related decline in TFQI_FT4_ levels in both women and men at ages < 50 and < 53, respectively, but a marked increase after that. Lower TSH levels were significantly associated with a higher prevalence and lower odds of having fewer TNs using multiple nodules as the base category in both men and women (both *P* for trend < 0.05). Additionally, lower TFQI_FT3_ and TFQI_FT4_ levels were significantly associated with a higher prevalence of TNs in women (both *P* for trend < 0.05), and lower TFQI_FT3_ levels were significantly associated with a higher prevalence of TNs in men. Both higher TFQI_FT3_ and TFQI_FT4_ levels were significantly associated with higher odds of having fewer TNs using multiple nodules as the base category in women. However, the relationships between TFQI_FT4_ and the prevalence or number of TNs in men were not found.

**Conclusions:**

The trends of THs, TSH, TFQI_FT4_, and TFQI_FT3_ at different ages were sex-dependent. Both TFQI_FT4_ and TFQI_FT3_ levels were negatively associated with the prevalence and number of TNs in women. The present results may lead to a better understanding of the sex-specific relationships between the development of the pituitary-TH axis and the formation of TNs.

## Introduction

Thyroid nodules (TNs) are local thyroid space-occupying lesions caused by various reasons (1). It was estimated that upwards of 67% of adults in the general population are found to possess one or more TNs varying widely by country (2, 3). Even though merely 1% to 5% of these nodules are malignant, they have garnered significant public interest owing to the psychological distress and anxiety they impose upon patients (4, 5). In addition, a substantial body of evidence has consistently shown a significantly higher prevalence of TNs in women compared to men (6, 7). Consequently, it becomes imperative to investigate the risk factors of TNs, specifically for each gender.

Negative feedback mechanisms play a crucial role in maintaining the homeostasis of thyroid hormones (THs) and thyrotropin (TSH) regulation (8). Nevertheless, recent research suggests a notable prevalence of elevated levels of both TSH and THs in euthyroid adults (9, 10). To address this, the thyroid feedback quantile-based index (TFQI) has been proposed as a means of assessing negative feedback of THs and reflecting the central sensitivity to THs within the general population, specifically targeting deviations from normal TH values rather than extreme ranges (11). The calculation of TFQI_FT4_ is based on the levels of FT4 and TSH as determined by a specific formula, whereas TFQI_FT3_ is computed by substituting FT4 with FT3 in the TFQI_FT4_ formula (12). A recent investigation demonstrated that the feedback indices exhibited a decline that varied according to sex as individuals aged in children and adolescents, and higher TFQI_FT4_ and TFQI_FT3_ values were related to a lower prevalence of TNs (13). Nevertheless, there is a lack of research examining the trend of TFQI_FT4_ and TFQI_FT3_ as individuals age, as well as the associations between these indices and the prevalence of TNs in euthyroid adults.

While there is a consensus that high TSH levels are a risk factor for thyroid cancer, a previous study has demonstrated that TSH levels were suppressed in young individuals with nodules (14). Furthermore, a recent study involving 2460 adults has indicated that lower TSH levels are related to a heightened risk of TNs compared with those with normal TSH levels (15). Thus, further studies including a larger number of participants on the associations between these indices and the prevalence of TNs in euthyroid adults were needed.

Taken together, the objective of this study was to investigate the trends of TSH, THs, and the feedback indices including TFQI_FT4_ and TFQI_FT3_ as individuals aged, and the associations of TSH, THs, and the feedback indices with the prevalence and the number of TNs in Chinese euthyroid adults in a sex-specific manner.

## Method

### Study design and population enrollment

**Figure 1:**
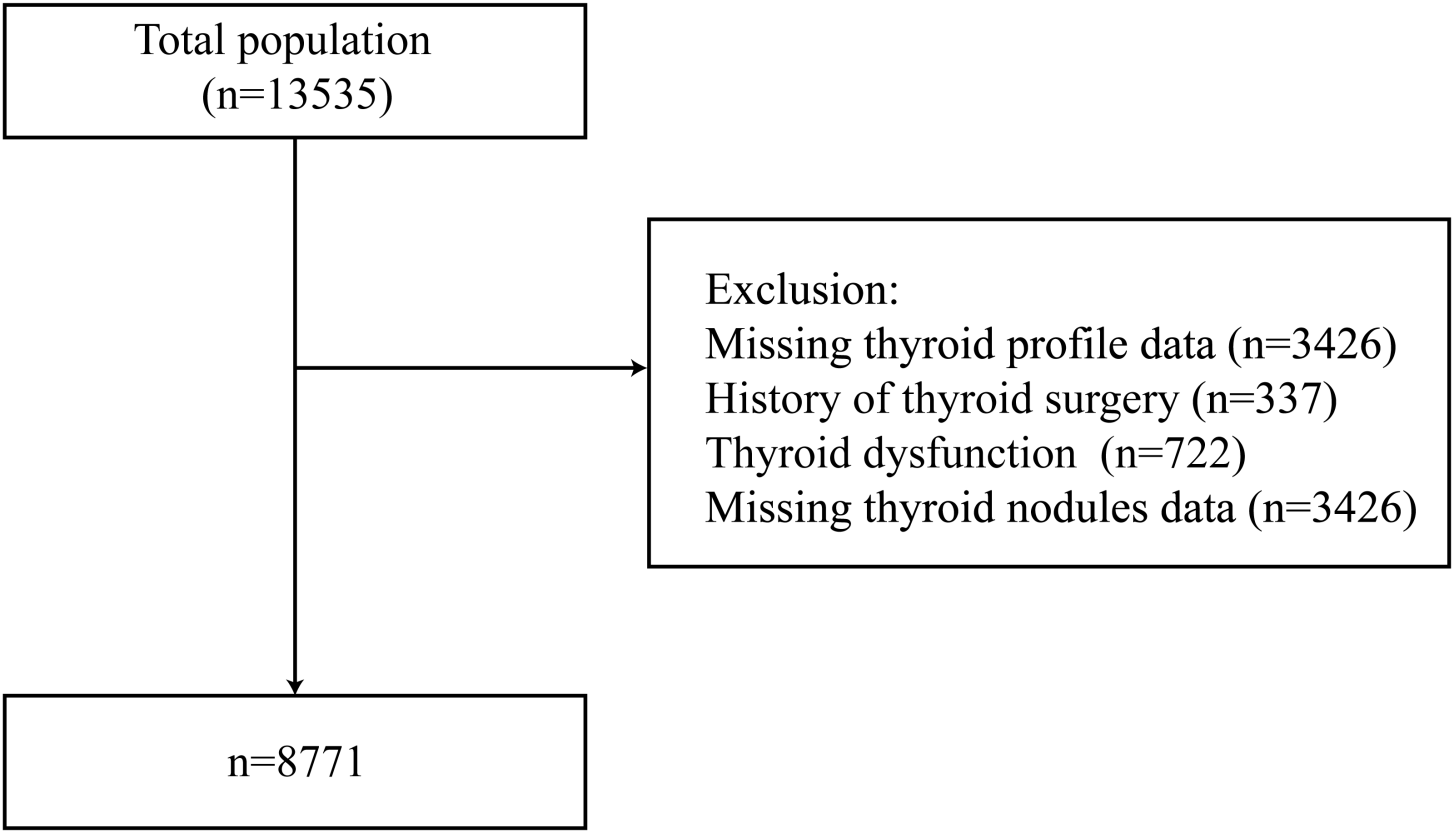
Flowchart of the study participants.

The participants were 13535 adults between November 2021 and September 2022 from ten study sites using a stratified cluster sampling method. The registration number is ChiCTR2100054130 (www.chictr.org). Specific methods are as previously described (12, 16). A flowchart for enrolment of study participants is shown in **Figure 1**. Exclusion criteria included missing thyroid profile values (n=3426), a history of thyroid surgery (n=337), thyroid dysfunction (n=722), and missing thyroid nodule data (n=3426). Thus, a total of 8771 participants were eligible for inclusion in the present study. All participants provided informed consent. The study was approved by the Ethics Committee of Shunde Hospital of Southern Medical University.

### Health and demographic characteristics

Age, sex, medication information, education level, disease history, medication information, smoking status, and drinking habits were self-reported. The body mass index (BMI) of the subjects was calculated as weight in kilograms divided by height in meters squared. An automated electronic device (HEM-752 FUZZY, Omron, China) was used to measure systolic (SBP) and diastolic blood pressure (DBP). Ultrasound was performed using B-mode US imaging (MX7, Mindray Shenzhen, P.R. China) with a 13 MHz linear array probe. A thyroid nodule was defined as present if the size of the nodule on ultrasound was found to be ≥2 mm by some highly trained technicians.

### Laboratory tests

Blood samples were taken from participants who had fasted for 10 hours between 7:00 a.m. and 10:00 a.m. The levels of FT3, FT4, TSH, thyroid peroxidase antibodies (TPOAb), and thyroglobulin antibody (TgAb) in the serum were analyzed using the Microparticle Enzyme Immunoassay (MEIA) technique on the UniCel Dxi 800 Access instrument (Beckman Coulter, USA). We used the normal reference ranges for thyroid function and thyroid antibodies recommended by manufacturers: TSH (0.56-5.91 pmol/L), FT3 (3.53-7.37 pmol/L), FT4 (7.98-16.02 pmol/L), TPOAb (0-9 IU/mL), and TgAb (0-4.9 IU/mL) (17, 18). Fasting plasma glucose (FPG), 2-h postprandial plasma glucose (PPG), creatinine, serum total cholesterol (TC), triglyceride (TG), high-density lipoprotein cholesterol (HDL-C), and low-density lipoprotein cholesterol (LDL-C) were measured by the automatic biochemical analyzer AU5831 (Beckman Coulter, USA). Glycated hemoglobin (HbA1c) levels were determined by high-performance liquid chromatography (HLC-723G8, TOSOH, Japan).

### Definitions of TFQI

TFQI_FT3_ was calculated as cumulative distribution function (cdf) FT3 - (1 - cdf TSH), and TFQI_FT4_ = (cdf) FT4 - (1 - cdf TSH). The value of TFQI is between -1 and 1. A lower value indicates higher thyroid hormone sensitivity (12, 19).

### Definitions of covariates

Baseline hypertension was defined as SBP ≥140 mmHg, DBP ≥90 mmHg, or self-reported use of antihypertensive medication (20). Diabetes was identified in individuals whose FPG level ≥7.0 mmol/L, PPG ≥11.1 mmol/L, HbA1c ≥6.5%, and/or having a self-reported diagnosis of diabetes as the previous study (21). The definition of thyroiditis was TPOAb > 9.0 IU/mL or TGAb > 4.9 IU/mL (22). Dyslipidemia was defined as TC ≥ 6.22 mmol/L, TG ≥ 2.26 mmol/L, LDL ≥ 4.14 mmol/L, or HDL < 1.04 mmol/L, or self-reported diagnosis of dyslipidemia as the previous study (23, 24). The abusive drink was defined as those with an alcohol consumption >30 g/day for men and >20 g/day for women (25). Smoking status was classified as never smoker, former smoker, and current smoker (26). Education level was categorized as less than a high school diploma, completed school graduate, and beyond high school (27). The Chronic Kidney Disease Epidemiology Collaboration (CKD-EPI) estimate of renal function was calculated using GFR = 141 × min(Scr/κ, 1)α × max(Scr/κ, 1)^-1.209^ × 0.993^Age^ × 1.018 [if woman] (28).

### Statistical analysis

Continuous variables were expressed as mean (standard deviation) or median (upper and lower quartiles), and categorical variables were expressed as numbers (%). These characteristics of the individuals with and without thyroid nodules were compared using analysis of the t-test, the Mann-Whitney U test, or the chi-square test.

A smooth curve was used to show the relationship between the thyroid function/thyroid sensitivity indices and age. Pearson correlation analysis was used to calculate the correlation between thyroid sensitivity indices and age. Multivariable logistic regression was used to identify the associations of the thyroid function/thyroid sensitivity indices with thyroid nodules, adjusted for age, BMI, eGFR, hypertension, thyroiditis, dyslipidemia, alcohol consumption, education, smoking status, and diabetes. No collinearity among the confounders was found (variance inflation factor < 5) (29). Multinomial logistic regression analysis was constructed to assess the associations of thyroid function/thyroid sensitivity indices with the thyroid nodule numbers (normal, solitary nodule, multiple nodules) using the fully adjusted model. The base category for the multinomial logistic regression is multiple nodules. *P* values <0.05 were considered statistically significant. The missing values were handled by multiple imputations using the mice R-package. All statistical analyses were conducted using R (version 4.2.1).

## Results

### General characteristics of the participants

**Table 1** describes the basic characteristics of participants. Among the 8771 participants, 5333 were women, and 3438 were men. In women, compared with participants without thyroid nodules, participants with thyroid nodules were more likely to be elders and low education obtained, had significantly higher BMI, SBP, DBP, FPG, HbA1c, PPG, TPOAb, TG, TC, and LDL-C (*P <*0.05), and lower eGFR, TFQI_FT3_, TFQI_FT4_, and TSH. In men, compared with the thyroid nodules- group, participants in the thyroid nodules+ group are more likely to be current smokers, had significantly higher ages, BMI, SBP, DBP, FPG, HbA1c, and PPG, and lower education level, FT3, TSH, FT3/FT4 ratio, TFQI_FT3,_ and eGFR.

**Table 1:** Basic characteristics of participants in the study.

	Women			Men		
	Nodules-	Nodules+	*P*	Nodules-	Nodules+	*P*
N	2690	2643	–	2210	1228	–
Age, year	43.35 (11.20)	51.34 (11.81)	<0.001	44.01 (11.60)	52.02 (12.51)	<0.001
Education, n (%)			<0.001			<0.001
less than high school	960 (35.69%)	1386 (52.44%)	–	652 (29.50%)	538 (43.81%)	–
high school	614 (22.83%)	578 (21.87%)	–	609 (27.56%)	298 (24.27%)	–
beyond high school	1116 (41.49%)	679 (25.69%)	–	949 (42.94%)	392 (31.92%)	–
Abusive drink, n (%)	18 (0.67%)	8 (0.30%)	0.085	156 (7.06%)	85 (6.92%)	0.935
Smoking, n (%)			0.500			<0.001
non-smokers	2673 (99.37%)	2619 (99.09%)	–	1471 (66.56%)	722 (58.79%)	–
former smokers	7 (0.26%)	9 (0.34%)	–	147 (6.65%)	120 (9.77%)	–
current smokers	10 (0.37%)	15 (0.57%)	–	592 (26.79%)	386 (31.43%)	–
BMI, kg/m^2^	22.81 (3.34)	23.40 (3.30)	<0.001	24.59 (3.50)	24.85 (3.28)	0.030
SBP, mmHg	118.46 (15.35)	124.38 (17.50)	<0.001	126.65 (16.02)	130.70 (18.21)	<0.001
DBP, mmHg	79.93 (20.34)	81.59 (10.66)	<0.001	84.36 (22.14)	85.86 (11.76)	0.01
FPG, mmol/L	4.78 (1.08)	5.04 (1.31)	<0.001	4.93 (1.50)	5.25 (1.85)	<0.001
HbA1c, %	5.63 (0.64)	5.81 (0.79)	<0.001	5.80 (0.88)	6.00 (1.05)	<0.001
PPG, mmol/L	7.79 (2.96)	8.45 (3.39)	<0.001	7.97 (3.80)	8.87 (4.37)	<0.001
FT3, pmol/L	5.21 (0.54)	5.20 (0.56)	0.567	5.58 (0.56)	5.50 (0.57)	<0.001
FT4, pmol/L	11.11 (1.37)	11.18 (1.41)	0.068	11.46 (1.45)	11.54 (1.42)	0.135
TSH, mIU/L	1.93 (1.37;2.68)	1.68 (1.17;2.39)	<0.001	1.71 (1.26;2.31)	1.59 (1.15;2.19)	<0.001
FT3/FT4 ratio	0.48 (0.07)	0.47 (0.07)	0.075	0.49 (0.08)	0.48 (0.07)	<0.001
TFQI_FT3_	-0.05 (0.39)	-0.12 (0.39)	<0.001	0.07 (0.38)	-0.01 (0.37)	<0.001
TFQI_FT4_	-0.02 (0.37)	-0.07 (0.38)	<0.001	-0.02 (0.38)	-0.04 (0.37)	0.221
TPOAb, IU/ml	1.10 (0.50;4.19)	1.20 (0.50;4.60)	0.019	0.80 (0.40;2.02)	0.80 (0.40;2.10)	0.157
TgAb, IU/ml	0.10 (0.07;0.64)	0.10 (0.07;0.64)	0.242	0.07 (0.07;0.10)	0.07 (0.07;0.20)	0.657
eGFR, mL/min/m^2^	101.67 (14.64)	95.25 (15.39)	<0.001	93.68 (15.26)	88.51 (16.33)	<0.001
TG, mmol/L	1.04 (0.78;1.48)	1.16 (0.83;1.69)	<0.001	1.46 (1.02;2.21)	1.44 (1.02;2.18)	0.86
TC, mmol/L	5.26 (2.32)	5.48 (1.06)	<0.001	5.37 (1.05)	5.41 (1.07)	0.412
HDL-C, mmol/L	1.54 (0.32)	1.53 (0.33)	0.432	1.30 (0.28)	1.30 (0.29)	0.876
LDL-C, mmol/L	2.83 (0.70)	3.01 (0.74)	<0.001	3.12 (0.75)	3.13 (0.76)	0.624

Continuous variables were expressed as mean (standard deviation) or median (upper and lower quartiles), and categorical variables were expressed as numbers (%). The P value was calculated by Student’s t-test, the Mann-Whitney U test, or the chi-squared test. A two-tailed P < 0.05 was considered significant.

BMI, body mass index; SBP, systolic blood pressure; DBP, diastolic blood pressure; FPG, fasting plasma glucose; HbA1c, Glycated hemoglobin; PPG, postprandial glucose; FT3, free triiodothyronine; FT4, free thyroxine; TSH, thyroid-stimulating hormone; FT3/FT4 ratio, FT3 to FT4 ratio; TFQI_FT3_, the thyroid feedback quantile-based index calculated by FT3; TFQI_FT4_, the thyroid feedback quantile-based index calculated by FT4; TPOAb, thyroid peroxidase antibody; TgAb, thyroglobulin antibody; eGFR, estimated glomerular filtration rate; TG, triglyceride; TC, total cholesterol; HDL-C, high-density lipoprotein-cholesterol; LDL-C, low-density lipoprotein-cholesterol.

### Characterization of central sensitivity to THs indices in women and men at different ages

**Figure 2:**
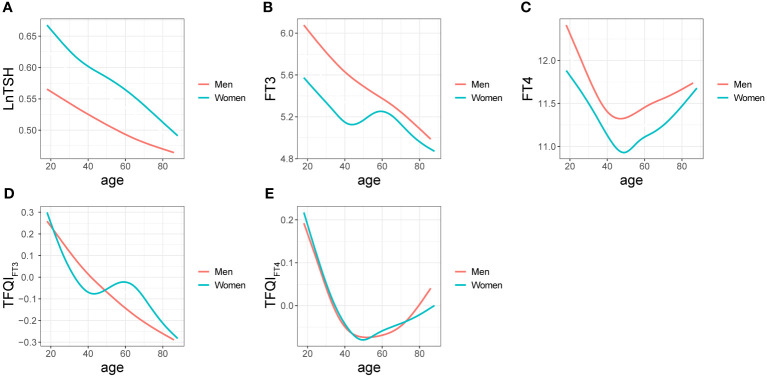
Relationships between age and LnTSH **(A)**, FT3 **(B)**, FT4 **(C)**, TFQI_FT3_ **(D)**, and TFQI_FT4_ **(E)** in men and women are denoted by curves.

A negative relationship between age and LnTSH was observed in both women and men (**Figure 2A**). FT3 and age showed a smooth and consistent negative correlation in men. At ages <47 and >59, a marked decrease in FT3 was observed in women, whereas an increase was observed at ages between 47 and 59 (**Figure 2B**). Regarding the relationship between age and FT4, apparent changes were observed at 48 and 47 years in women and men, respectively (**Figure 2C**). Prominent smooth suppression of TFQI_FT3_ was observed in men aged 18-88 years and in women aged < 43 and > 59 years (**Figure 2D**). There was an age-related decline in TFQI_FT4_ levels in both women and men at ages < 50 and < 53, respectively, but a marked increase after that (**Figure 2E**).

As shown by the Spearman coefficients in **Table 2**, negative relationships between age and thyroid function levels such as LnTSH, FT3, FT4, TFQI_FT3_, and TFQI_FT4_ were observed in both women and men (*P <*0.01, all).

**Table 2:** Spearman’s coefficient between each parameter and age.

	Women		Men	
	r	*P*	r	*P*
LnTSH	-0.055	<0.001	-0.049	0.004
FT3	-0.068	<0.001	-0.320	<0.001
FT4	-0.068	<0.001	-0.063	<0.001
TFQI_FT3_	-0.077	<0.001	-0.268	<0.001
TFQI_FT4_	-0.086	<0.001	-0.084	<0.001

### Associations of thyroid sensitivity indices with the prevalence of thyroid nodules

**Figure 3:**
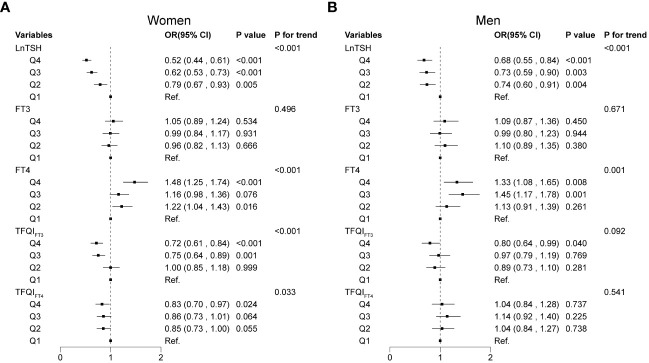
Associations of thyroid sensitivity indices with thyroid nodules using multivariable logistic regression models. Associations of thyroid sensitivity indices with thyroid nodules in women **(A)** and men **(B)**.

**Figure 3** summarizes the results of the sex-specific associations (OR, 95% CI) between quartiles of thyroid sensitivity indices and the odds of thyroid nodules. The multivariable logistic regression adjusted for age, BMI, eGFR, smoking status, education, alcohol consumption, thyroiditis, dyslipidemia, hypertension, and diabetes. In women, participants in the three higher quartiles of LnTSH, TFQI_FT3_, and TFQI_FT4_ were less likely to have thyroid nodules compared with the lowest quartile group (Q1) of LnTSH, TFQI_FT3_, and TFQI_FT4_, respectively (*P*<0.05, all). In men, participants in Q4 of LnTSH and TFQI_FT3_ were less likely to develop thyroid nodules, compared with Q1 of LnTSH and TFQI_FT3_, respectively (both *P* < 0.05). Compared with participants in Q1, the prevalence of thyroid nodules increased by 48% and 33% in the fourth quartile (Q4) of FT4 in women (OR 1.48; 95% CI [1.25, 1.74]) and men (OR 1.33; 95% CI [1.08, 1.65]), respectively. However, the associations between FT3 and the prevalence of thyroid nodules were not found in either women or men nor were the associations between TFQI_FT4_ and thyroid nodules in men.

### Associations of thyroid sensitivity indices with normal/solitary nodule/multiple nodules category

**Figure 4:**
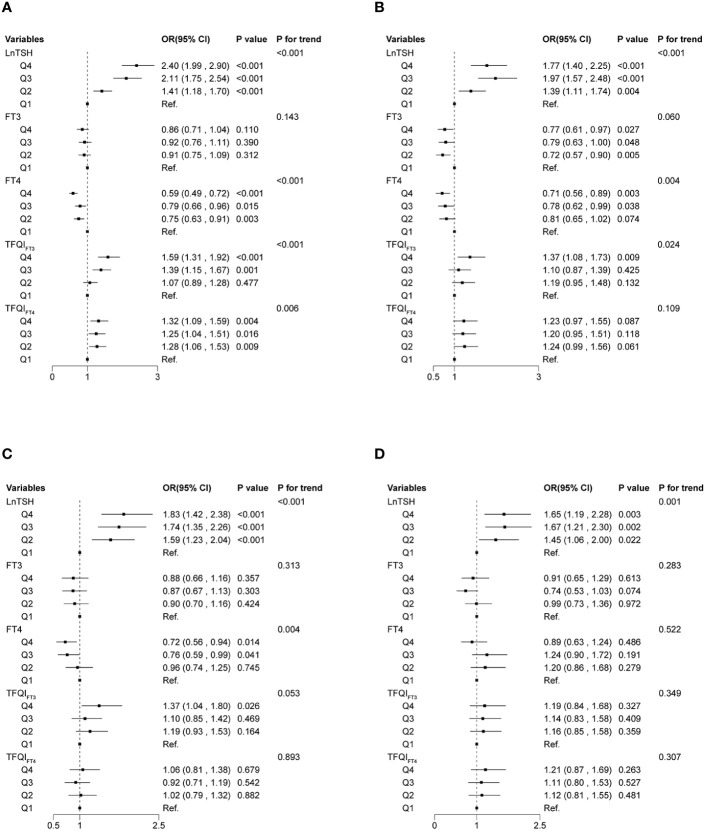
Associations of thyroid sensitivity indices with thyroid nodules using multinomial logistic regression models. Normal vs. multiple nodules **(A)** and solitary nodules vs. multiple nodules **(B)** in women. Normal vs. multiple nodules **(C)** and solitary nodules vs. multiple nodules **(D)** in men. The base category for the multinomial logistic regression is multiple nodules.

In the multinomial logistic regression (**Figure 4**), compared with the first quartile of LnTSH, the fourth quartile of LnTSH was associated with higher odds of being normal or having a solitary nodule in both women [normal vs. multiple nodules: OR 2.40, 95% CI (1.99, 2.90); solitary nodule vs. multiple nodules: OR 1.77, 95% CI (1.40, 2.25)] and men [normal vs. multiple nodules: OR 1.83, 95% CI (1.42, 2.38); solitary nodule vs. multiple nodules: OR 1.65, 95% CI (1.19, 2.28)] (*P* < 0.05). The results were similar for both TFQI_FT3_ and TFQI_FT4_ in women (*P* < 0.087). Participants in Q4 of FT4 were less likely to be normal or have a solitary nodule in women and less likely to be normal in men. Participants in Q4 of FT3 were less likely to have a solitary nodule in women only (*P* < 0.05).

### Sensitivity analysis

We performed a sensitivity analysis to assess the robustness of our findings using data excluding participants with missing values. The results remained similar and significant (**Supplementary Figure S1**-**S3**).

## Discussion

To the best of our knowledge, the current study first presented the TFQI_FT4_ and TFQI_FT3_ trends in relation to age, as well as their associations with the prevalence and quantity of TNs, within a substantial cohort of euthyroid adults in China. Our findings indicate a general decline in TFQI_FT3_ among both genders, except women aged 43 to 59 years, where it increased slightly. Additionally, TFQI_FT4_ exhibited a decline until the age of 48 and 47 in both men and women, followed by a slight increase. Lower TSH levels were significantly associated with a higher prevalence in both men and women. Additionally, lower TFQI_FT3_ and TFQI_FT4_ levels were significantly associated with a higher prevalence in women, and lower TFQI_FT3_ levels were significantly associated with a higher prevalence of TNs in men. No associations were found between TFQI_FT4_ levels and the prevalence and number of TNs in men. These findings offer potential insights into the risk factors contributing to sex differences in TNs or other diseases related to the central sensitivity to THs. Prevention efforts were suggested to focus on thyroid hormone-sensitive individuals, particularly women and younger people.

In the current study, we found that the prevalence of TNs was found to be lower in men compared to women, which is consistent with the results in the previous studies. One meta-analysis including 102 entries showed that the prevalence of TNs was higher in women (36.51%) compared to men (23.47%) (6). Another study including 6,985,956 healthy Chinese participants who received health examinations found that the prevalence of TNs in women (age-standardized prevalence: 45.2% [95% CI, 44.1%-46.4%]) was significantly greater than that in men (age-standardized prevalence 31.2% [95% CI, 30.1%-32.2%]) (7). Previous studies have demonstrated that estrogen could exert a significant influence on the sex-specific prevalence of thyroid nodules through its proliferative effects rather than polymorphisms (30, 31). Estrogen could stimulate growth and simultaneously inhibit the differentiation of thyroid nodule-derived stem/progenitor cells, which may be the origin of thyroid nodules in women (31, 32).

Similarly to a previous study that included euthyroid participants aged 6-20 years (13), TSH and FT3 levels were found to be predominantly higher in male participants and decreased with age in the current study. In addition, we also revealed the sex-specific trend in TSH, THs, and feedback indices as individuals aged. Particularly, FT_3_ increased slightly among women aged 43 to 59 years, whereas it declined in men. As individuals progress in age, the daily synthesis of FT4 diminishes, yet the serum concentration of FT4 remains constant owing to the diminished activity of deiodinase and the reduced conversion of FT3 (33). Consequently, FT4 experiences a decline until the age of 50 in both genders, followed by a marginal increase as observed in the present study. Furthermore, the heightened levels of serum FT3 during perimenopause can be interpreted as a compensatory and adaptive mechanism to counteract fat accumulation and enhance energy expenditure amidst metabolic dysregulation (34). This hypothesis was substantiated by the finding that FT3 levels decreased after weight loss in the previous study (35). An alternative mechanism underlying the association between thyroid hormones and age or gender could be the dynamic changes in the expression of these hormones throughout the lifespan, responding to the changing needs of different organs and the aging process. However, these hypotheses lack sufficient evidence and necessitate further investigation and research.

While it is widely acknowledged that TSH plays a significant role in promoting nodule development and hastening nodule growth in adults (36), the relationship between TSH and TNs in euthyroid individuals remains inconclusive. One previous study reported TSH was not correlated with thyroid nodule formation (37), however other studies found that lower TSH levels were associated with a higher prevalence of TNs both in adolescents (13) and adults (15), which is consistent with the results in our study. As recommended in the 2023 European Thyroid Association Clinical Practice Guidelines for thyroid nodule management, the use of thyroid hormone to decrease TSH should be discouraged in the absence of elevated TSH (2). This is due to its lack of efficacy in adequately reducing the size of symptomatic nodules (38, 39). Interestingly, in the current study, we reported that lower TSH levels are associated with a higher number of TNs in both men and women. Thus, exploring whether TSH inhibition therapy can increase the number of TNs is a topic worth investigating in the future.

Previously, research on the associations between TFQI_FT4_ and TFQI_FT3_ and the prevalence of TNs in euthyroid adults was limited. A recent study found a negative relationship between TFQI_FT4_ and TFQI_FT3_ and the prevalence of TNs in children and adolescents (13). We observed similar associations in euthyroid women, but there was no significant association between TFQI_FT4_ and the prevalence of TNs in men. This difference in results may be partly due to the higher prevalence of TNs in women compared to men. Additionally, the levels of TFQI_FT4_ and TFQI_FT3_ may vary by sex, which could also contribute to the sex difference observed.

The current study possesses several limitations. Firstly, the cross-sectional design employed in this study precludes the establishment of longitudinal or causal relationships between the developmental alterations in the pituitary-thyroid hormone axis and the prevalence of TNs. Therefore, additional prospective cohort investigations or Mendelian randomization research are necessary to investigate the causal inference and further the realization of its clinical application. Secondly, despite our diligent efforts to control for various confounding factors, it is crucial to acknowledge the potential influence of residual and recall bias on the estimation of effects. Thirdly, the lack of further investigation into the classification of benign and malignant nodules hinders our ability to investigate the associations between these indices and the malignancy of TNs.

In conclusion, the trends of FT3, FT4, TSH, TFQI_FT4_, and TFQI_FT3_ in relation to age were sex-dependent. Both TFQI_FT4_ and TFQI_FT3_ levels were negatively associated with the prevalence and number of TNs in women. However, the associations of TFQI_FT4_ with prevalence and number of TNs in men were not found. The present results may lead to a better understanding of the sex-specific relationships between the development of the pituitary-TH axis and the formation of TNs, and may partially explain the higher incidence of TNs in women than in men.

## Data availability statement

The raw data supporting the conclusions of this article will be made available by the authors, without undue reservation.

## Ethics statement

The studies involving humans were approved by the Ethics Committee of the Shunde Hospital of Southern Medical University. The studies were conducted in accordance with the local legislation and institutional requirements. The participants provided their written informed consent to participate in this study.

## Author contributions

YL: Data curation, Writing – original draft. GY: Methodology, Project administration, Writing – original draft. NY: Investigation, Writing – original draft. SL: Project administration, Software, Writing – original draft. DW: Project administration, Writing – original draft. QM: Project administration, Writing – original draft. LL: Conceptualization, Writing – review & editing. HW: Conceptualization, Writing – review & editing. JS: Supervision, Writing – review & editing.
