# Supplementary material for: Sex-specific associations between the developmental alterations in the pituitary-thyroid hormone axis and thyroid nodules in Chinese euthyroid adults: a community-based cross−sectional study
Source: Front Endocrinol (Lausanne). 2024 May 10;15:1379103. doi: 10.3389/fendo.2024.1379103 (PMC11116631; doi:10.3389/fendo.2024.1379103)
Supplement: Supplementary file 1 [file DataSheet_1.docx]

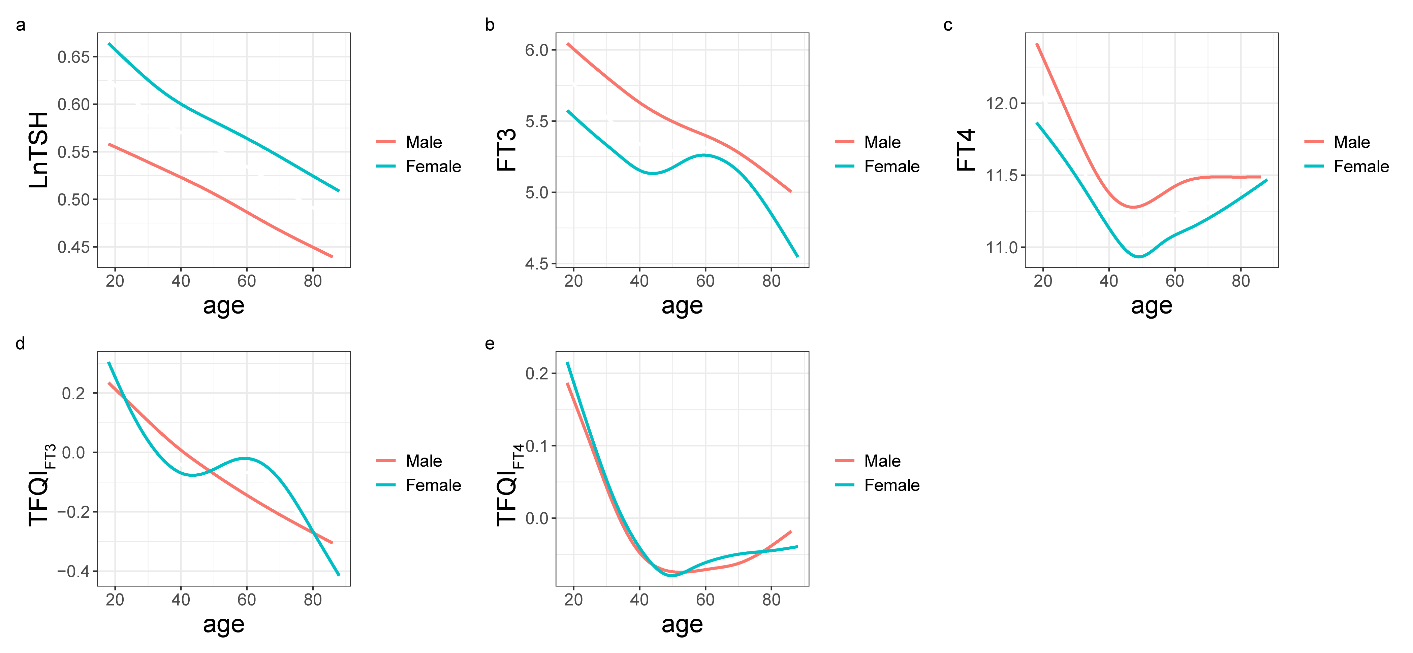


**Figure S1.** Relationships between age and LnTSH (a), FT3 (b), FT4 (c), TFQI_FT3_ (d), and TFQI_FT4_ (e) in men and women are denoted by curves.


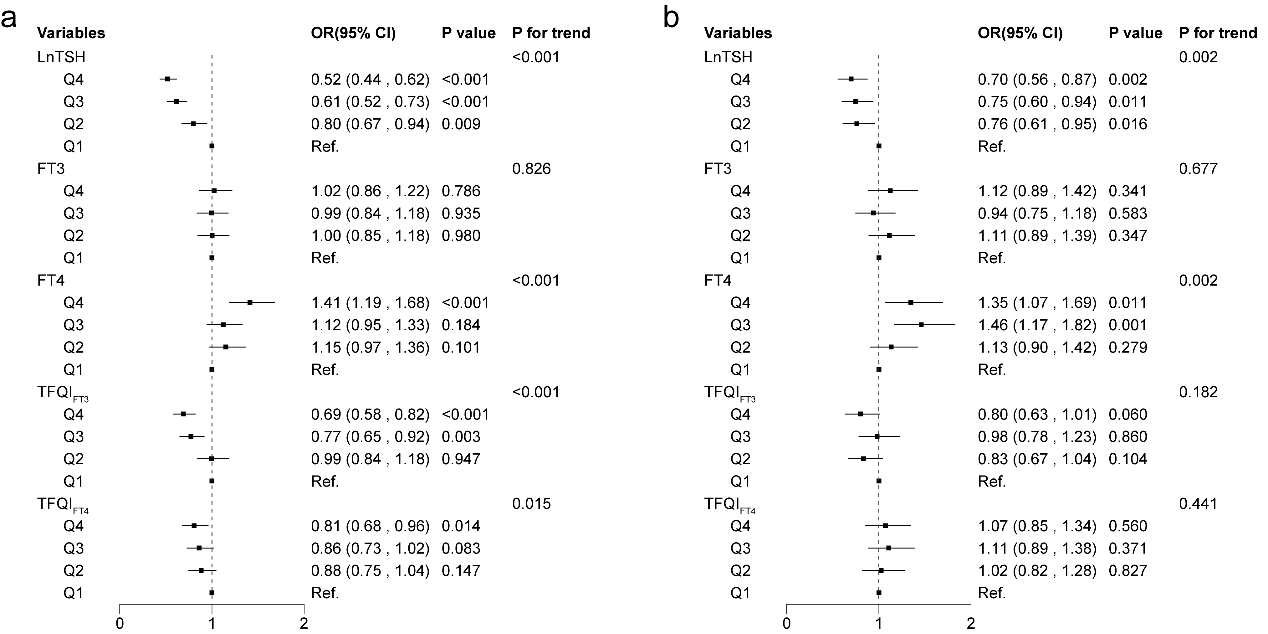


**Figure S2.** Associations of thyroid sensitivity indices with thyroid nodules using multivariable logistic regression models.

Associations of thyroid sensitivity indices with thyroid nodules in women (a) and men (b).


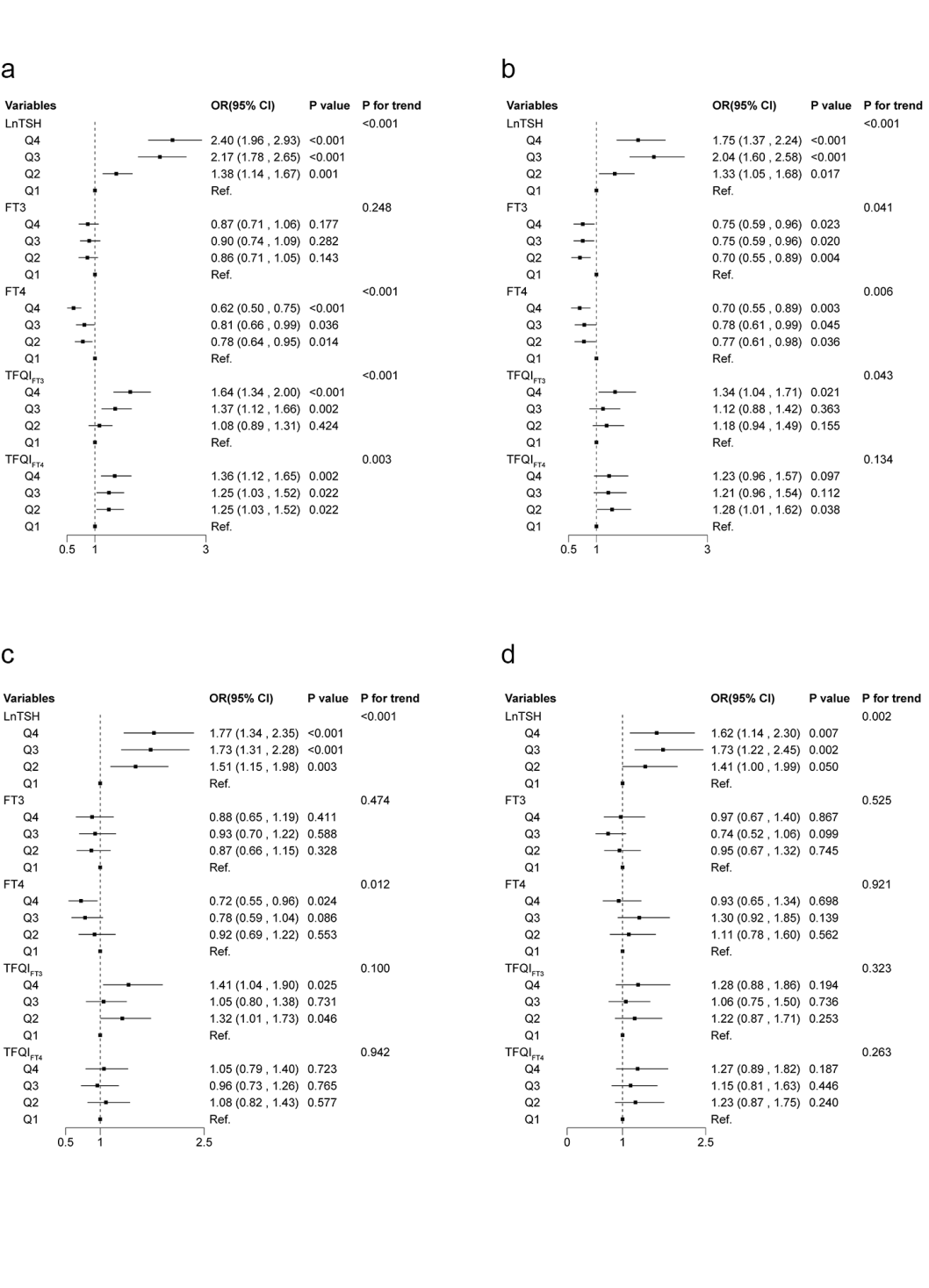


**Figure S3.** Associations of thyroid sensitivity indices with thyroid nodules using multinomial logistic regression models.

Normal vs. multiple nodules (a) and solitary nodule vs. multiple nodules (b) in women. Normal vs. multiple nodules (c) and solitary nodule vs. multiple nodules (d) in men.

The base category for the multinomial logistic regression is multiple nodules.
